# Treating Diabetes Mellitus: Pharmacophore Based Designing of Potential Drugs from* Gymnema sylvestre* against Insulin Receptor Protein

**DOI:** 10.1155/2016/3187647

**Published:** 2016-02-28

**Authors:** Mohammad Uzzal Hossain, Md. Arif Khan, S. M. Rakib-Uz-Zaman, Mohammad Tuhin Ali, Md. Saidul Islam, Chaman Ara Keya, Md. Salimullah

**Affiliations:** ^1^Department of Biotechnology and Genetic Engineering, Life Science Faculty, Mawlana Bhashani Science and Technology University, Santosh, Tangail 1902, Bangladesh; ^2^Department of Genetic Engineering and Biotechnology, Life Science Faculty, Shahjalal University of Science and Technology, Kumargaon, Sylhet 3114, Bangladesh; ^3^Department of Biochemistry and Molecular Biology, University of Dhaka, Dhaka 1000, Bangladesh; ^4^National Institute of Biotechnology, Ganakbari, Ashulia, Savar, Dhaka 1349, Bangladesh; ^5^Department of Biology and Chemistry, North South University, Bashundhara, Dhaka 1229, Bangladesh; ^6^Molecular Biotechnology Division, National Institute of Biotechnology, Ganakbari, Ashulia, Savar, Dhaka 1349, Bangladesh

## Abstract

Diabetes mellitus (DM) is one of the most prevalent metabolic disorders which can affect the quality of life severely. Injectable insulin is currently being used to treat DM which is mainly associated with patient inconvenience. Small molecules that can act as insulin receptor (IR) agonist would be better alternatives to insulin injection. Herein, ten bioactive small compounds derived from* Gymnema sylvestre* (*G. sylvestre*) were chosen to determine their IR binding affinity and ADMET properties using a combined approach of molecular docking study and computational pharmacokinetic elucidation. Designing structural analogues were also performed for the compounds associated with toxicity and less IR affinity. Among the ten parent compounds, six were found to have significant pharmacokinetic properties with considerable binding affinity towards IR while four compounds were associated with toxicity and less IR affinity. Among the forty structural analogues, four compounds demonstrated considerably increased binding affinity towards IR and less toxicity compared with parent compounds. Finally, molecular interaction analysis revealed that six parent compounds and four analogues interact with the active site amino acids of IR. So this study would be a way to identify new therapeutics and alternatives to insulin for diabetic patients.

## 1. Background

Diabetes mellitus (DM) is a group of metabolic diseases in which there are high blood sugar levels or hyperglycemia over a prolonged period. Type 2 diabetes mellitus (T2DM) is the most prevalent (95%) one compared to type 1 (T1DM) [1]. In case of type 1 body fails to produce insulin, whereas in type 2 body shows resistance to insulin. Also, alteration of many genes and their products may contribute in T2DM [2, 3]. DM is one of the prime concerns of morbidity and mortality around the globe with an expected projection of 366 million cases in 2030 compared to 171 million in 2000 [4]. Among the cases, the city dwellers of developing countries are likely to be the most affected group [4]. Diabetes is a common scenario in the South and East Asia Region predominantly in Bangladesh, India, Sri Lanka, Bhutan, Mauritius, and Maldives. In 2013, Saquib et al. reported that there are nearly 72 million adult diabetics in these countries which will be raised to 123 million by 2035 [5]. The very same cross-sectional study conducted among 402 middle class Bangladeshi citizens also reveals that as much as 35% of them have T2DM and 45% have metabolic complications [5].

Chronic hyperglycemia in DM leads to the damage of kidneys, nervous system, eyes, heart, and blood vessels [6]. Acute myocardial infarction and fatal reinfarction are common with diabetic patients in the acute phase and during the first year of follow-up [7–11]. However, to compensate the lower level of insulin in blood, administration of insulin injection followed by intensive treatment with multidose subcutaneous insulin can reduce up to 30% of mortality in diabetic patients with acute myocardial infarction [12–14].

Insulin, secreted from the pancreatic *β*-cells, is the most studied peptide hormone for its importance to control glucose homeostasis. This 51-aa hormone binds to its transmembrane *α*_2_ *β*_2_ glycoprotein insulin receptor (IR). Intracellular tyrosine kinase domain of IR is activated by insulin binding and triggers cascade of intracellular signaling pathways which eventually enhance cellular uptake of glucose from circulation [15, 16]. Diabetes mellitus (DM) is the complication caused either by the poor tissue responsiveness to insulin or by the metabolic malfunction of insulin production. Type 1 DM is an autoimmune disorder where pancreatic *β*-cell is destroyed by the immune system which causes insufficient insulin secretion, whereas type 2 DM is caused by resistance of body tissue towards insulin [17, 18]. In both cases, insulin injection is necessary to maintain glucose homeostasis. Although recombinant insulin is now available and the technology for insulin delivery has improved in the recent past but the discomfort and inconveniency associated with insulin injection cannot be denied. Additionally, long term insulin injection leads to the development of cutaneous complications such as allergy, lipoatrophic area development, and injection abscesses [19]. Therefore, the discovery of small molecules that are capable of mimicking insulin action and can be administered orally would be better alternative therapies for the diabetic disorder. As of now very little work has been done to develop such kind of small molecules. Moreover, the identified small molecules have several problems such as poor IR specificity or bioavailability. For instance, arylalkylamine vanadium salts are strong IR activators but associated with questionable bioavailability, and demethylasterriquinone B1 (DAQ B1) has insulin mimicking potential but associated with significant cytotoxicity [20, 21].

Herein, we selected* G. sylvestre* derived small compounds to check their significance to be assigned as potential candidate for IR activators designing.* G. sylvestre* is a commonly seen herb in South Asian region (India, Sri Lanka, and Bangladesh as well) which is considered as promising source of antidiabetic compounds.* G. sylvestre* derived compounds demonstrated considerable antidiabetic results in several experiments [22]. Experimental trials have been conducted in animal models with* G. sylvestre* active constituents of which insulin-like activity and mechanism of action of hypoglycemic principles have been investigated in detail [23]. Amid many, Gymnemic acids III, IV, V, and VII and Gymnemoside B were recognized as antihyperglycemic active constituents while Conduritol A was found to have cataract-suppressing effect [24]. Moreover, it has been reported that another active ingredient from* G. sylvestre* leaves extract, GS4, plays a very significant role in the regulation of both type 1 and type 2 diabetes [24, 25].

Very few studies have been conducted on plant derived compounds with pharmacoinformatics elucidation for the identification of novel therapeutics against diabetes [26, 27]. In this study, we have considered antidiabetic compounds from* G. sylvestre* and their analogues as possible drug candidates for insulin receptor. Various computational tools have been used to perform pharmacoinformatics validations of these compounds as well as to investigate their structural (similar and different) analogues [28–36]. Also, Computer Aided Drug Design (CADD) tools have been used for the structural property as well as identification of interaction between insulin receptor and candidate drugs [28, 32–35, 37–40]. Finally, AutoDock tools were utilized for the assessment of ligand-protein interaction [38, 39]. This inclusive* in silico* analysis may provide insights into the understanding of drug-protein interaction and to design effective antidiabetic drugs.

## 2. Materials and Methods

**Figure 1:**
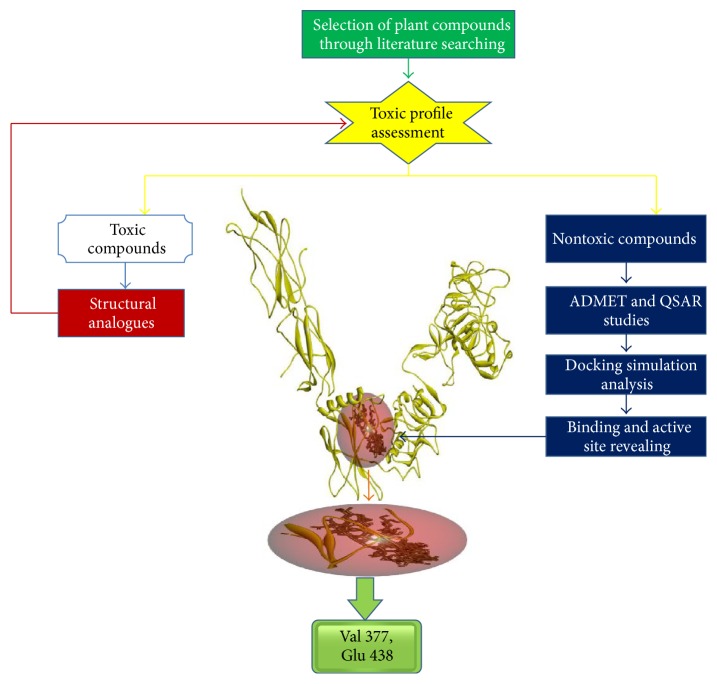
Schematic representation of the study.

The outline of this study was shown in Figure 1.

### 2.1. Preparation of Target Protein and Selection of Novel Drugs

**Figure 2:**
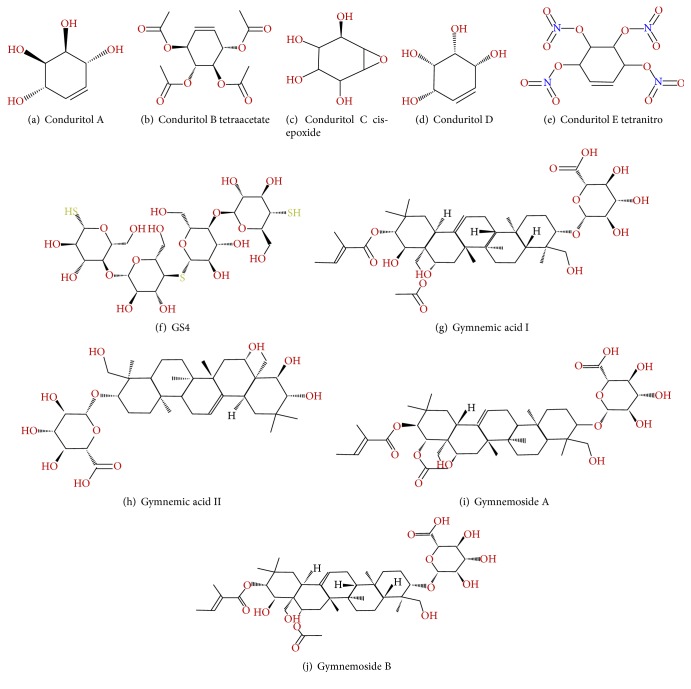
Two-dimensional structures of the ten candidate drugs from* Gymnema sylvestre*.

The 3D (three-dimensional) crystal structure of insulin receptor protein 3LOH used in this study was retrieved from RCSB (Research Collaboratory for Structural Bioinformatics) Protein Data Bank (PDB) [28]. The ligands from PDB files of 3LOH were removed using Discovery Studio 4.0 client (http://accelrys.com/products/discovery-studio/). The polypeptide chains (A, B, C, and D) of 3LOH were removed, and only E chain (insulin binding domain, ectodomain) of* Homo sapiens* was saved as PDB file format for further analysis (see Supplementary Figure S1 in Supplementary Material available online at http://dx.doi.org/10.1155/2016/3187647). After removal of the ligands and chains (A, B, C, and D), chain E of insulin receptor 3LOH was effectively used to screen and find out novel molecules for the treatment of diabetes. For the selection of drugs, existing literatures and databases were searched for plants having antidiabetic properties supported by laboratory study for itself and/or its compounds [23–25, 41, 42]. Among many,* Gymnema sylvestre*, a plant common in South Asia, were selected for the current study. A total of ten compounds (Figure 2) were selected from* Gymnema sylvestre* based on the following criteria: (i) tested compounds having antidiabetic (T2DM) effect (Gymnemoside B and Conduritol A) and their analogues (Gymnemoside A and Conduritol B tetraacetate, Conduritol C cis-epoxide analogue, Conduritol D, and Conduritol E tetranitro), (ii) tested compounds with no antidiabetic effect (Gymnemic acid I, Gymnemic acid II), and (iii) one unique compound, GS4, having antidiabetic effects against both types (T1DM and T2DM) [24, 25]. The two-dimensional (2D) structure of the selected active compounds of this plant was retrieved from Click2drug (http://www.click2drug.org/), zinc database (http://zinc.docking.org/) and PubChem compound [29] as SDF (Structure Data File) format. Afterwards, the SDF format of the compounds was converted to PDB file by using the Open Babel [30]. The 3D structure optimization of these compounds was done by the ACD/Chemsketch [31].

### 2.2. Pharmacoinformatics Elucidation

Different computational tools and web databases were exploited for the pharmacoinformatics elucidation of active compounds that might have the potentiality to exhibit insulin-like activity by interacting with the insulin receptor protein. The pharmacophoric library screening, ADMET (absorption, distribution, metabolism, excretion, and toxicity) and QSAR (quantitative structural-activity relationship) properties were carried out by Osiris property explorer [32], Molinspiration [33], AcTor [34], admetSAR [35], and ACD/I-Lab [36]. Briefly, Osiris property explorer [32] computes various drug related properties like tumorigenicity, mutagenicity, irritation, reproductive effect, drug likeness, and drug-score prediction based on chemical structure. Molinspiration offers broad range of cheminformatics software tools that support molecule manipulation and fragmentation, calculation of various molecular properties such as QSAR, molecular modeling, and drug design [37]. The ACToR (Aggregated Computational Toxicology Resource) database, that locates many types and sources of data, has information about* in vitro* bioassays and* in vivo* toxicology assays on chemical structure derived from more than 150 sources [34]. The admetSAR (absorption, distribution, metabolism, excretion, and toxicity structure-activity relationship database) [35] and ACD/I-Lab [36] handle existing ADMET-associated information from the available literature. Default parameters of these online servers are used for the pharmacoinformatics analysis.

### 2.3. Design New Drugs

To improve the antidiabetic activity of the four tested drugs, novel compounds were designed by generating their analogues (Supplementary Figure S2). ACD/Chemsketch [31], Discovery Studio 4.0 clients, and Open Babel [30] were employed to accomplish the task.

### 2.4. Energy Minimization of Designed Molecules

In designed structures, an accurate alignment of the side chains is an indispensable requirement in order to link the structural gap and encompass loop rearrangements, secondary structure elements, repacking of core residues, and so forth. Here, We have calculated the free relative binding energies for these complexes using the YASARA (Yet Another Scientific Artificial Reality Application) force field [38]. YASARA uses a full atomic description of designed molecules, whose parameters have been optimized to minimize the damage done to designing procedure. The YASARA energy function includes terms that have been found to be important for molecules stability [39, 40, 43].

### 2.5. Active Site Analysis

For the identification of the active site and determination of pocket of the insulin receptor, Computed Atlas of Surface Topography of proteins (CASTp) (http://sts.bioe.uic.edu/castp/calculation.php) server was used. This server provides an online resource for locating, delineating, and measuring concave surface regions on three-dimensional structures of proteins including pockets located on protein surfaces and voids buried in the interior of proteins [44].

### 2.6. Molecular Docking Analysis

Before initiating the docking simulations, polar hydrogen was added to insulin receptor protein and prepared as PDBQT files in AutoDock tools [45]. To prepare for the ligand-protein interaction analysis, the torsional bonds were set free from the ligands and saved as PDBQT file format. The prepared crystal structure of 3LOH_E was covered with grid box parameter for observing its interaction with drug molecules. For this, a grid box encompassing each ligand was set with the dimension (*X* = 98, *Y* = 98, and *Z* = 98) and center (*X* = 10.303, *Y* = 107.879, and *Z* = 38.693) as well as grid box spacing 1.0 Å for blind docking analysis. All the docking calculations were then performed with the set parameters by using AutoDock Vina [46] which is an automated procedure for predicting the interaction of ligands with biomacromolecular targets. The visualization of protein-ligand interaction was performed by Pymol 1.1 [47] and Discovery studio 4.0 client.

## 3. Results

### 3.1. Pharmacophore Study of Drug Compounds and Their Analogues

The pharmacophore and QSAR properties of the compounds were analyzed for their drug likeness, drug score, toxicity, structural polarity, and oral bioavailability by using various software mentioned in Section 2 and the results are listed in Tables 1 and 2. Among the ADMET properties, human intestinal absorption is highest for Conduritol B tetraacetate (0.9899 out of 1.0) while Caco-2 permeability is highest for Gymnemic acid I and Gymnemoside A (0.9223 out of 1). All the other compounds show moderate to very high human intestinal absorption (0.5133–0.9691) as well as Caco-2 permeability (0.5225–0.9040). No drug seems to cross the blood brain barrier as revealed by their score ranging from 0.5000 to 0.9473. Unbound fraction of the drugs in plasma is also very high (48–100%) except for Conduritol E tetranitro (11%) implying their well distribution among the tissues. As expected, blood brain distribution of the drugs is less than zero except Conduritol E tetranitro (0.33). Very high metabolic score (>0.8000) was observed for all the ten candidate drugs. However, out of ten candidate drugs, Conduritol A, Conduritol B tetraacetate, Conduritol C cis-epoxide, and Conduritol D showed mutagenic, reproductive, irritating, and tumorigenic effect, respectively (Table 1). Ligand properties were found in acceptable range for all the tested drugs (Table 2). However, the six compounds possess better ligand properties in contrast to the four drugs (Conduritols A–D) showing toxicity. The six compounds require less docking energy (−8.0 to −10.9 Kcal/mol) compared to the four toxic (−5.2 to −5.7 Kcal/mol) compounds. With broader polar surface area (220–401) six compounds show stronger interactions to the target by donating two times more (7.7 versus 3.5 on average) and accepting three times more (14.7 versus 4.75) hydrogen ion than that of the four toxic compounds. In addition to this, oral bioavailability of the six drugs is more than 70% with one exception of Gymnemic acid I (30–70%). Furthermore, the six compounds are more likely to be used as drugs as revealed by their drug likeness and drug score.

**Figure 3:**
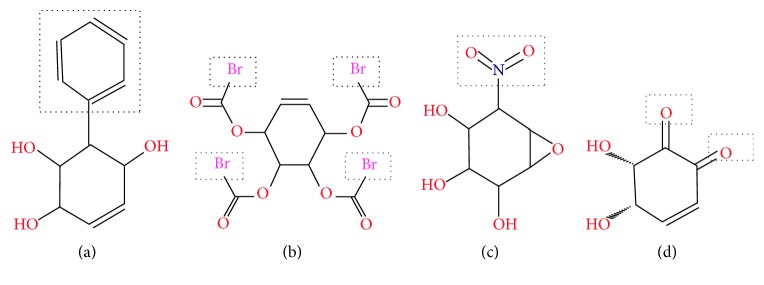
New designed analogues of Conduritol A (a), Conduritol B tetraacetate (b), Conduritol C cis-epoxide (c), and Conduritol D (d). To increase their binding affinity, analogues have been generated by adding benzene, Br^−^, NO_2_^−^, and O_2_^−^ into their 2D structures, respectively.

**Figure 4:**
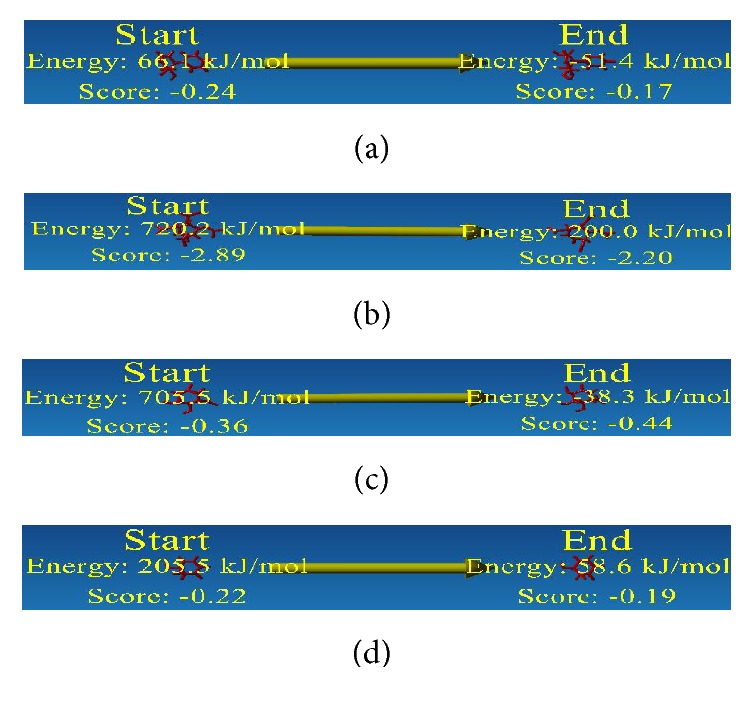
Energy minimization structure of designed molecules. Here red sticks designate the designed molecules. Designed analogues of Conduritol A (a), Conduritol B tetraacetate (b), Conduritol C cis-epoxide (c), and Conduritol D (d).

**Figure 5:**
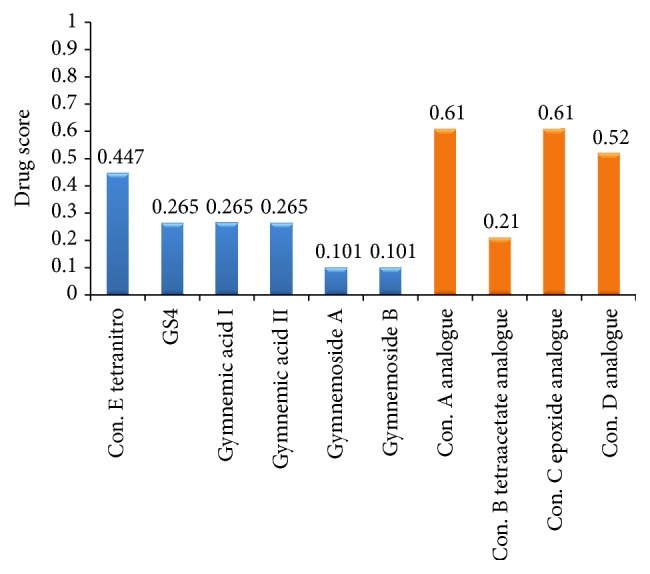
Computational drug score of suggested drug molecules. Here blue color indicates the medicinal plant derived drug compounds and orange color indicates the designed analogue drug compounds.

The four drugs showing good ligand and ADMET properties but some toxic effects and lower binding affinity for the insulin receptor have been considered for further analysis with an aim of identification of novel drug candidates. As a consequence, ten structural analogues for each of the four drugs were generated and analyzed for pharmacophore and QSAR properties (Supplementary Figure S2, Table 3). Among the 40 designed analogues, best one for each drug, namely, Conduritol A with benzene, Conduritol B tetraacetate with Br^−^, Conduritol C cis-epoxide with NO_2_^−^, and Conduritol D with O_2_^−^ side chain (Figure 3), was selected based on their pharmacophore and QSAR properties (Table 3). Remarkably, the newly designed four analogues showed all the pharmacophore and QSAR properties within acceptable range. Before the docking energy analysis, YASARA program was used in refining physical realism, stereochemistry, and side-chain accurateness in designed molecules. This program yields the energy (START versus END) that ensures accuracy of designed molecules (Figure 4). The energy comparison among designed molecules from the START energy (66.1 KJ/mol, 720.2 KJ/mol, 705.5 KJ/mol, and 205.5 KJ/mol) to END energy (−51.4 KJ/mol, 200.0 KJ/mol, −38.3 KJ/mol, and 58.6 KJ/mol) confirm the stability of the structural complexes of the designed molecules during the minimization of energy. The docking energy for the new analogues was found lower (−7.5 to −8.1 Kcal/mol) in comparison to their parent compound (−5.2 to −5.7 Kcal/mol) as well as no toxic effect was associated with them. Finally, the four analogues with improved drug likeness and drug score (Figure 5) also claimed their position in the queue of the novel antidiabetic drugs.

### 3.2. Active Site and Molecular Docking Analysis

**Figure 6:**
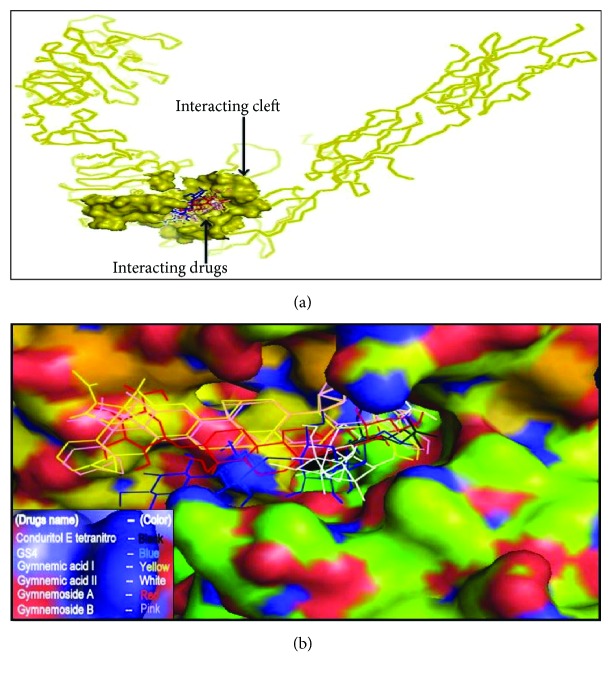
Docking simulation of six compounds of* Gymnema sylvestre* with E chain of 3LOH. (a) Ribbon structure of 3LOH_E showed the binding affinity of all six drugs to the E chain. (b) Space filling model also showed the six drugs in active site cleft of E chain.

**Figure 7:**
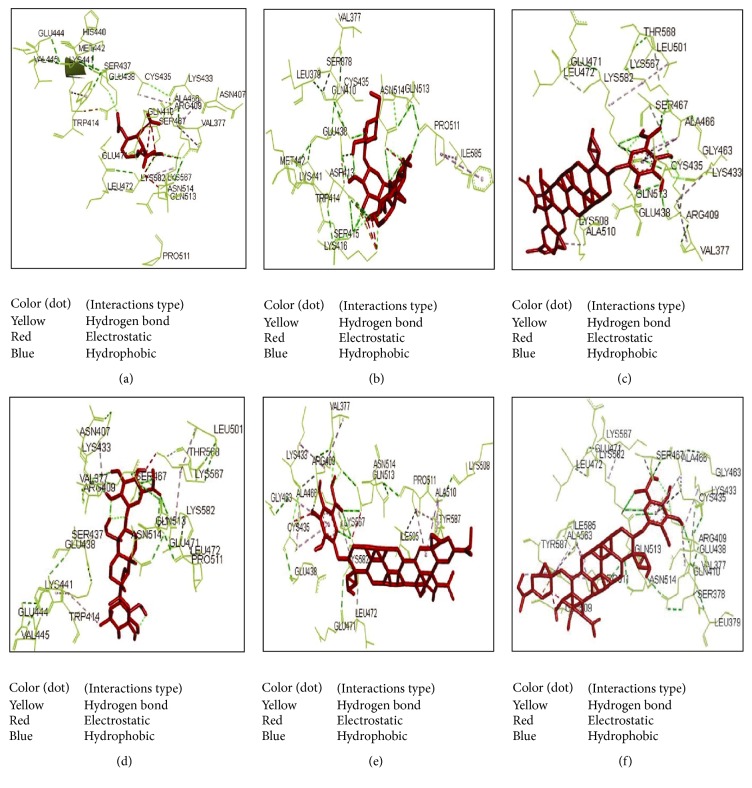
Interaction between drugs (a–f) and active site amino acid residues of 3LOH_E. (a) Conduritol E tetranitro, (b) GS4, (c) Gymnemic acid I, (d) Gymnemic acid II, (e) Gymnemoside A, and (f) Gymnemoside B. Red stick represents the drug compounds. Yellow dots represent the hydrogen bond interaction. Red dots represent the electrostatic interactions and blue dot also represents the hydrophobic interactions with amino acid residues. The common interactions with amino acid residues among the six compounds are Val 377, Glu 438, and Leu 472.

**Figure 8:**
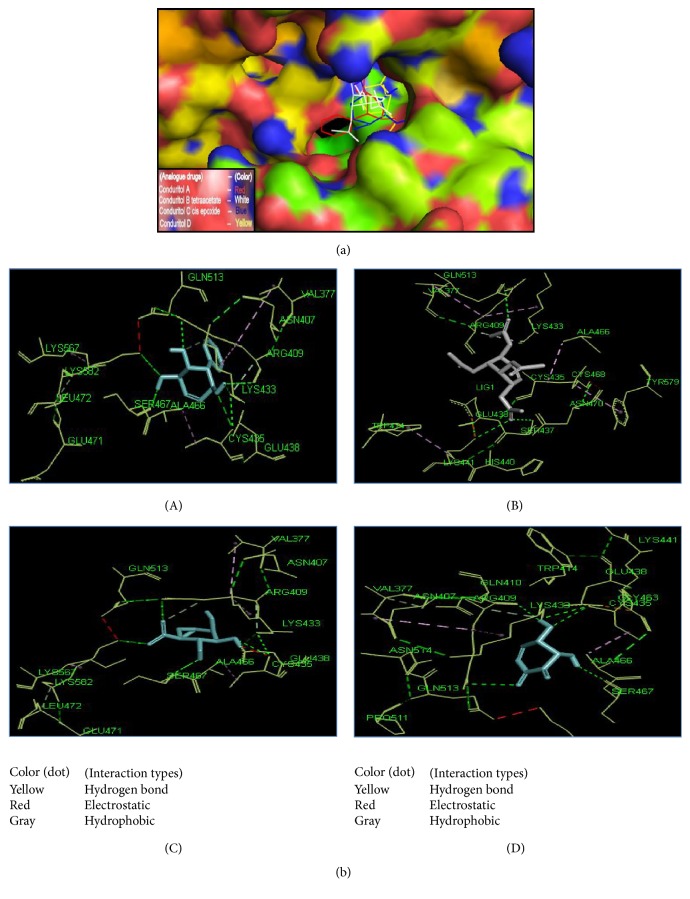
Interaction between the four novel designed molecules and active site amino acid residues of 3LOH_E. (a) Space filling model of 3LOH_E with four novel designed molecules in the active site cleft of E chain. (b) Designed drug compounds interactions with amino acid residues of insulin receptor chain E. (A) Conduritol A analogue, (B) Conduritol B tetraacetate analogue, (C) Conduritol C cis-epoxide analogue, and (D) Conduritol D analogue. Here, marine sticks designate the drug compounds. The interactions with most common amino acid residues of these novel molecules are Val 377, Lys 433, Cys 435, Glu 438, and Ala 466.

**Figure 9:**
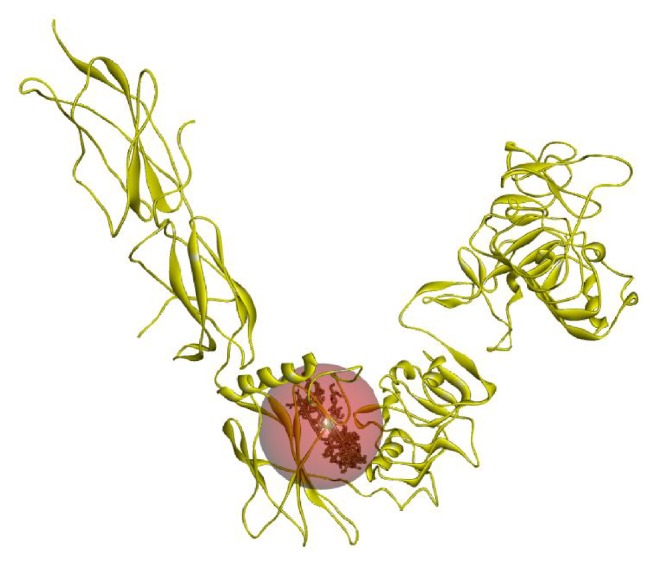
Drugs (natural plant compounds and designed molecules) binding into the binding site of 3LOH_E chain. Val 377 and Glu 438 were the common binding residues among these drug compounds.

The active site area of the insulin receptor protein and the number of amino acid residues involved in it were determined with the CASTp server. This provides a significant insight of the docking simulation study to locate the active site cleft and the amino acid residues that interact with different ligands. The preeminent active site is found with 5311.9 area and a volume of 40219 amino acids (Supplementary Figure S3). The molecular docking analysis performed with AutoDock Vina for the six candidate drugs showing good pharmacophore and QSAR properties revealed variations in their binding energies. AutoDock Vina produced nine possible binding positions as output for each drug. Out of nine possible ligands binding positions the best one was chosen for each compound based on the lowest docking energy. The results pointed out that all the six compounds efficiently bind to insulin receptor 3LOH_E (Figure 6). The amino acid interactions of insulin receptor 3LOH_E with these drugs were also identified (Figure 7). Amino acids that were unanimous to interact with at least five compounds were adopted as interacting amino acid. It was observed that Val 377, Arg 409, Lys 433, Cys 435, Glu 438, Glu 471, Leu 472, and Pro 511 exhibited strong binding interactions with the six docked compounds (Figure 7). Among these, Val 377, Glu 438, and Leu 472 were common in interactions between IR protein and all the six drugs. The interactions that play significant role in the determination of binding energy and stability of these receptor-ligand complexes were recognized as hydrogen bond and hydrophobic and electrostatic interactions. Evidently, all of the nine interacting amino acids are located in the active site pocket of the insulin receptor protein (Supplementary Figure S3). Besides these drugs, the docking results of the structural analogues of the remaining four drugs confirmed their strong interactions with the insulin receptor protein (Figure 8). Val 377, Arg 409, Lys 433, Cys 435, Glu 438, Ala 466, Ser 467, Glu 471, and Leu 472 were found in binding interactions with the four designed analogues (Figure 8). But Val 377, Lys 433, Cys 435, Glu 438, and Ala 466 were common in interactions with all designed drugs and 3LOH_E. Interestingly, these amino acids are also in the active site pocket of the insulin receptor protein (Supplementary Figure S3). These amino acid residues identified a binding site within the E chain of 3LOH by blind docking analysis (Supplementary Figure S4). Interacting amino acids with docking analysis confirmed that all the drug molecules for this study bind in the same binding site (Figure 9). It is therefore to predict that amino acid residues Val 377 and Glu 438 could play a crucial role for both plant derived drug compounds and designed drug molecules in mimicking the insulin activity.

## 4. Discussion

Compounds screening leading to drug design have been an active area of research for many years. Due to the wearisome and expensive nature of investigational screening procedures, computational compound screening has been pursued extensively in recent years [48, 49]. The endeavor of this study was based on the relationship between drug-receptor interactions and its* in silico* analysis for designing, identifying, and evaluating novel drugs against diabetes mellitus. The new drug candidates were identified from the medicinal plant* G. sylvestre* abundant in South Asia to study them as potent antidiabetic drugs (Figure 2). The target for the current study is insulin receptor protein “3LOH” of which 3D crystal structure is available with ligands. To prepare the target for the screening of drug compounds, we removed the ligands and chains (A, B, C, and D) and kept only E chain of the 3LOH crystal structure (Supplementary Figure S1). Blind docking analysis for the whole 3LOH_E protein with the candidate drugs discloses their (drugs) highest affinity towards the active site cleft amino acid residues of the 3LOH_E chain (Supplementary Figure S3). Henceforth, the drugs affinity towards IR protein's active site could be shown which is the most desired binding sites for the suggested drug candidates (Supplementary Figure S4).

The pharmacophore properties of the selected molecules were carried out by using the up-to-date online-based and license-agreed software. The pharmacokinetics and pharmacology for absorption, distribution, metabolism, elimination, and toxicity are assessed by the ADMET properties that describe the disposition of a drug-like compound within the body of an organism [50]. All the candidate drugs tested in this study show moderate to high absorption rate. Importantly no drug seems to cross blood brain barrier. High distribution of the drugs in plasma indicates their availability in all tissues. However, no blood brain distribution is observed. Metabolism of all the drugs is shown to be perfect. With no toxicity six drugs, namely, Conduritol E tetranitro, GS4, Gymnemic acid I, Gymnemic acid II, Gymnemoside A, and Gymnemoside B show characteristics suitable as drug (Table 1).

Investigational analysis revealed that the putative compounds (Figure 2) used in the present analysis have significant values to diabetes mellitus. All the ten selected drug compounds were analyzed on the basis of binding energy values to insulin receptor and their drug properties (Table 2). After docking study, all six compounds with no toxic effects showed their high binding affinity to the E chain of the insulin receptor protein (Figure 6). Gymnemoside A and Gymnemoside B were interacted with the highest number (21) of amino acids. Although GS4 possess the ability to interact with its target having the highest number of hydrogen donors as well as acceptor, the highest binding energy was observed in Gymnemoside A (−10.9 Kcal/mol). It is clearly apparent that Conduritol E tetranitro, GS4, Gymnemic acid I, Gymnemic acid II, Gymnemoside A, and Gymnemoside B might be suggested as potent antidiabetic drug candidates because of having lowest docking energy indicating their higher binding affinity to the insulin receptor protein. To impart this study towards availability and improvement of antidiabetic drugs, the four compounds, namely, Conduritol A, Conduritol B tetraacetate, Conduritol C cis-epoxide, and Conduritol D showing lower binding affinity to insulin receptor and toxic effects were employed to add benzene, Br^−^, NO_2_^−^, and O_2_^−^ in their structure to generate novel compounds and to increase their antidiabetic potentiality (Figure 3). The pharmacophore and the QSAR analysis suggest that there is no indication of toxicity of these designed molecules and hence allow them to be the probable antidiabetic drug candidates (Table 3). The docking study of these novel compounds identifies their higher binding affinity to their target insulin receptor. Interactions of the native plant derived compounds as well as four analogues are exerted in the active site amino acid of the IR (Figures 6, 7, and 8). The interactions that play significant role in the determination of binding energy and stability of these receptor-ligand complexes were recognized as hydrogen bond and hydrophobic and electrostatic interactions. This interaction of these drugs confirm their insulin mimicking properties. Considering all these facts, it might be assumed that these drugs will activate IR and facilitate translocation of glucose transporter 4 (Glut-4) to the plasma membrane and influx of glucose and thereby help maintain normal blood glucose level [15]. Therefore, all the six drug compounds especially Gymnemoside A, B and Gymnemic acid I, II together with four designed drug molecules might be proposed as oral drugs after experimental validation against diabetes mellitus.

This* in silico* analysis supported the potentials of tested antihyperglycemic natural compounds (Gymnemoside B and GS4) as oral drugs [24, 25]. Along with this, this study ascertained the potentials of nonconfirmed antihyperglycemic natural compounds (Gymnemoside A, Conduritol E tetranitro, Gymnemic acid I, and Gymnemic acid II) as oral drugs. Finally, this study proposes four novel drugs for their experimental validation as potential oral antidiabetic drug (Figure 3).

The current study shed light into the interactions between 3D structure of insulin receptor active site and predicted drug molecules. All the pharmacophore analysis including molecular docking of candidate drug molecules reveals their high affinity to the IR active site amino acids and their eligibility as bioavailable oral drugs. Though these* in silico* analyses predicted ideal antidiabetic drugs it is limited by the lack of experimental validations. In addition to this, no comparison studies among the candidate drugs as well as candidate drugs with known antidiabetic drugs have been performed. Nevertheless, the results of this study provide opportunities for further evaluation of the proposed drugs* in vitro* and* in vivo* for establishing their candidature as alternative oral drugs for the treatment of diabetes.

## 5. Conclusion

Prolonged usage of insulin often leads to insulin insensitivity and insulin resistance of the cell as well as other daunting side effects such as the risk of hypoglycemia. Moreover, the complications and pain associated with the administration of insulin cannot also be denied. Taking these complications into consideration, in this present study, using computational and bioinformatics tools we have identified, designed, and proposed ten oral based novel therapeutic drugs for the treatment of diabetes mellitus which might help reduce our dependency on insulin without causing considerable side effects and associated pain during administration. Further wet lab assessment of these drugs has to be performed to establish their insulin mimicking activity.

## Supplementary Material

Only E chain ( Insulin binding domain, ectodomain) among the polypeptide chains (A,B,C,D & E) of 3LOH insulin receptor of Homo sapiens was saved as PDB file format for insulin mimicking analysis (Supplementary figure S1). The ligands from PDB files of 3LOH as well as polypeptide chains were removed by using Discovery Studio 4.0 client (http://accelrys.com/products/discovery-studio/). Forty novel compounds were designed by generating analogues from the four tested drugs (Supplementary figure, S2) to improve the antidiabetic activity. ACD/Chemsketch [1], Discovery studio 4.0 client (http://accelrys.com/products/discovery-studio/) and open Babel [2] were employed to generate the analogues. The preeminent active site is found within 5311.9 area and a volume of 40219 amino acids into the E chain of 3LOH receptor (Supplementary figure, S3). These analyses were determined with the CASTp server (http://sts.bioengr.uic.edu/castp/). The binding site within the E chain of 3LOH was detected by Discovery Studio 4.0 client (http://accelrys.com/products/discovery-studio/) (Supplementary figure, S4).

## Figures and Tables

**Table 1:** ADMET properties of *Gymnema sylvestre* compounds. Most data of ADMET properties were measured according to a measuring scale of 1.0.

Properties	Conduritol A	Conduritol B tetraacetate	Conduritol C cis-epoxide	Conduritol D	Conduritol E tetranitro	GS4	Gymnemic acid I	Gymnemic acid II	Gymnemoside A	Gymnemoside B
Absorption										
Blood brain barrier	0.5000	0.9473	0.5337	0.5000	0.8859	0.5951	0.5396	0.5145	0.5396	0.5000
Human intestinal absorption	0.9305	0.9899	0.6488	0.9305	0.9691	0.9375	0.5405	0.6906	0.5405	0.5133
Caco-2 permeability	0.5225	0.5742	0.6220	0.5225	0.5443	0.8205	0.9223	0.8955	0.9223	0.9040

Distribution										
Blood brain distribution (LogBB)	−0.03	−0.01	−0.04	−0.03	0.33	−2.0	−0.48	−2	−0.46	−0.48
Fraction unbound in plasma	0.99	0.6	0.99	0.99	0.11	1	0.48	0.56	0.46	0.48
Volume of distribution (*V*_*d*_) (L/kg)	0.72	1.12	0.67	0.72	1.43	0.49	0.25	0.25	0.25	0.25

Metabolism										
CYP450 2C9 substrate	0.8335	0.8268	0.8081	0.8335	0.8564	0.8169	0.8585	0.8541	0.8585	0.8582
CYP450 2C9 inhibitor	0.8562	0.9772	0.9286	0.8562	0.7918	0.8666	0.8443	0.8549	0.8443	0.8346

Toxicity										
Mutagenicity	Yes	No	No	No	No	No	No	No	No	No
Tumorigenicity	No	No	No	Yes	No	No	No	No	No	No
Irritating effects	No	No	Yes	No	No	No	No	No	No	No
Reproductive effects	No	Yes	No	No	No	No	No	No	No	No

**Table 2:** Ligand properties of *Gymnema sylvestre* compounds.

Ligand properties	Conduritol A	Conduritol B tetraacetate	Conduritol C cis-epoxide	Conduritol D	Conduritol E tetranitro	GS4	Gymnemic acid I	Gymnemic acid II	Gymnemoside A	Gymnemoside B
Docking energy (Kcal/mol)	−5.2	−5.7	−5.3	−5.6	−8.0	−8.7	−10.8	−9.4	−10.9	−10.4
Molecular weight (g/mol)	146.1412	314.28792	162.1406	146.1412	326.1314	714.78	806.98	682.838	806.9757	806.9757
Number of H donor	4	2	4	4	2	14	7	9	7	7
Number of H acceptor	4	8	5	2	12	21	14	12	14	14
Log *S*	−1.958	−0.295	−0.237	−0.326	−4.065	−1.133	−1.133	−1.133	−5.38	−5.38
*c*Log *P*	−1.63	0.31	−0.205	−1.631	−2.829	−5.169	−5.169	−5.169	−2.976	−2.976
TPSA	80.92	105.2	93.45	80.92	220.2	401	401	401	229.7	229.7
Drug likeness	−0.05	−0.02	−0.218	−0.495	−2.168	−7.992	−7.992	−7.992	−6.524	−6.619
Oral bioavailability	30%–70%	30%–70%	30%–70%	30%–70%	More than 70%	More than 70%	30%–70%	More than 70%	More than 70%	More than 70%

**Table 3:** ADMET and QSAR properties of designed molecules.

Ligand properties	Conduritol A analogue	Conduritol B tetraacetate analogue	Conduritol C epoxide analogue	Conduritol D analogue
IUPAC name	3-Phenylcyclohex-5-ene-1,2,4-triol	Cyclohex-5-ene-1,2,3,4-tetrayl tetracarbonobromidate	5-Nitro-7-oxabicyclo[4.1.0]heptane-2,3,4-triol	(5S,6S)-5,6-Dihydroxycyclohex-3-ene-1,2-dione

Docking energy	−8.1 kcal/mol	−7.8 kcal/mol	−7.5 kcal/mol	−8.0 kcal/mol

Molecular weight	206.24	573.77	191.14	142.11

Number of hydrogen bond donors	3	2	3	2

Number of hydrogen bond acceptors	3	8	7	4

Solubility	−1.96	−5.87	−3.69	−0.43

TPSA	60.69	105.2	131.28	74.6

Number of rotatable bonds	1	8	1	1

Oral bioavailability	More than 70%	30%–70%	30%–70%	More than 70%

Absorption rate (Ka) min^−1^	0.022	0.054	0.0011	0.005

Volume of distribution (*V*_*d*_) (L/kg)	1.28	1.47	0.36	0.65

Violation	0	0	0	0

Toxicity	No	No	No	No

Drug likeness	−1.01	−2.36	−2.29	−2.97

Binding residues of 3LOH_E	Val 377, Asn 407, Arg 409, Lys 433, Cys 435, Glu 438, Ala 466, Ser 467, Glu 471, leu 472, Gln 513, Lys 567, and Lys 582	Val 377, Arg 409, Trp 414, Lys 433, Cys 435, Ser 437, Glu 438, Lys 441, His 440, Ala 466, Cys 468, Asn 470, and Gln 513, Tyr 579	Val 377, Asn 407, Arg 409, Lys 433, Cys 435, Glu 438, Ala 466, ser 467, Glu 471, leu 472, Gln 513, Lys 567, and Lys 582	Val 377, Asn 407, Arg 409, Lys 433, Cys 435, Glu 438, Lys 441, Gly 463, Ala 466, ser 467, Glu 471, leu 472, Pro 511, Gln 513, Asn 514, Lys 567, and Lys 582

## Abbreviations

DM: Diabetes mellitus
T1DM: Type 1 diabetes mellitus
T2DM: Type 2 diabetes mellitus
IR: Insulin receptor
CADD: Computer Aided Drug Design
Con.: Conduritol
3D: 3-dimensional
PDB: Protein data bank
ADMET: Absorption, distribution, metabolism, excretion, and toxicity
Log*S*: Logarithm of solubility
*c*Log*P*: Logarithm of partition coefficient
TPSA: The polar surface area
QSAR: Quantitative structural-activity relationship
*V*_*d*_: Volume of distribution
IUPAC: International Union of Pure and Applied Chemistry.
