# Supplementary material for: Treating Diabetes Mellitus: Pharmacophore Based Designing of Potential Drugs from Gymnema sylvestre against Insulin Receptor Protein
Source: Biomed Res Int. 2016 Feb 28;2016:3187647. doi: 10.1155/2016/3187647 (PMC4806669; doi:10.1155/2016/3187647)
Supplement: Supplementary file 1 — Only E chain ( Insulin binding domain, ectodomain) among the polypeptide chains (A,B,C,D & E) of 3LOH insulin receptor of Homo sapiens was saved as PDB file format for insulin mimicking analysis (Supplementary figure S1). The ligands from PDB files of 3LOH as well as polypeptide chains were removed by using Discovery Studio 4.0 client (http://accelrys.com/products/discovery-studio/). Forty novel compounds were designed by generating analogues from the four tested drugs (Supplementary figure, S2) to improve the antidiabetic activity. ACD/Chemsketch [1], Discovery studio 4.0 client (http://accelrys.com/products/discovery-studio/) and open Babel [2] were employed to generate the analogues. The preeminent active site is found within 5311.9 area and a volume of 40219 amino acids into the E chain of 3LOH receptor (Supplementary figure, S3). These analyses were determined with the CASTp server (http://sts.bioengr.uic.edu/castp/). The binding site within the E chain of 3LOH was detected by Discovery Studio 4.0 client (http://accelrys.com/products/discovery-studio/) (Supplementary figure, S4). [file 3187647.f1.docx]

**Supplementary materials:**

Only E chain ( Insulin binding domain, ectodomain) among the polypeptide chains (A,B,C,D & E) of 3LOH insulin receptor of *Homo sapiens* was saved as PDB file format for insulin mimicking analysis **(Supplementary figure S1)**. The ligands from PDB files of 3LOH as well as polypeptide chains were removed by using Discovery Studio 4.0 client (<http://accelrys.com/products/discovery-studio/>). Forty novel compounds were designed by generating analogues from the four tested drugs **(Supplementary figure, S2)** to improve the antidiabetic activity. ACD/Chemsketch [1], Discovery studio 4.0 client (<http://accelrys.com/products/discovery-studio/>) and open Babel [2] were employed to generate the analogues. The preeminent active site is found within 5311.9 area and a volume of 40219 amino acids into the E chain of 3LOH receptor **(Supplementary figure, S3).** These analyses were determined with the CASTp server (<http://sts.bioengr.uic.edu/castp/>). The binding site within the E chain of 3LOH was detected by Discovery Studio 4.0 client (<http://accelrys.com/products/discovery-studio/>) **(Supplementary figure, S4)**.

**Supplementary references:**

1. Y.-M. Fan, M.-S. Cheng, and Y.-X. Cheng, “The reformation inour organic chemistry classes caused by the excellent software ACD/ChemSketch,” *Journal of Guangzhou University (Natural Science Edition)*, vol. 6, p. 12, 2002.
2. N. M. O’Boyle, M. Banck, C. A. James, C. Morley, T. Vandermeersch,and G. R. Hutchison, “Open Babel: an open chemical toolbox,” Journal of Cheminformatics, vol. 3, article 33, 2011.

**Supplementary Figure:**


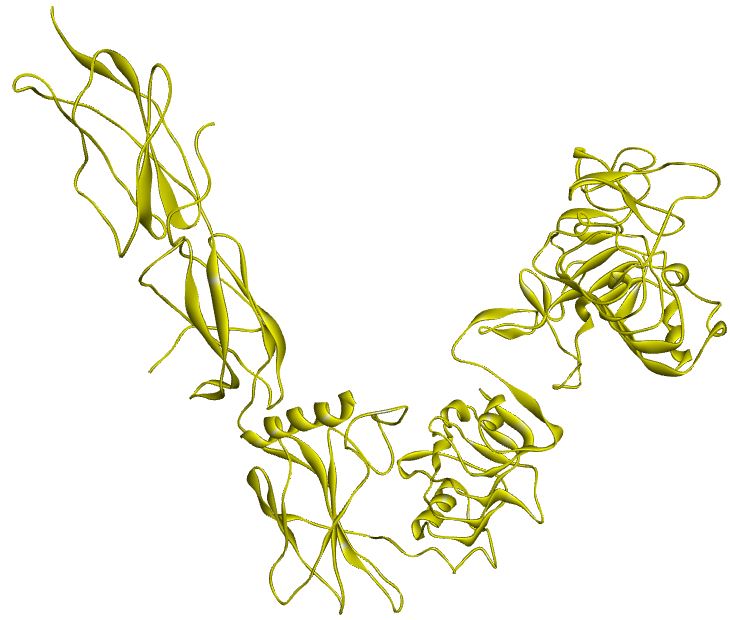


**Figure: Supplementary, S1: Modified 3D crystal structure of insulin receptor protein E chain of 3LOH without ligand.**

**1. Conduritol A**

1.
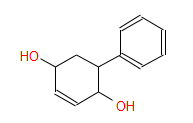
2.
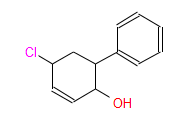
3.
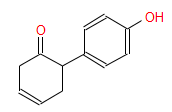


4.
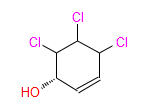
5.
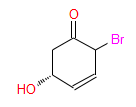
6.
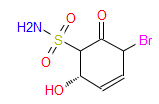


7.
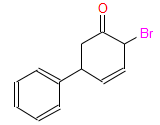
8.
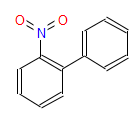
9.
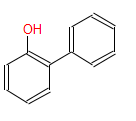


10.
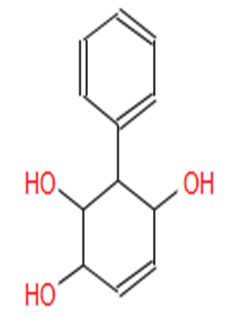


**2. Conduritol B tetraacetate**

1**.
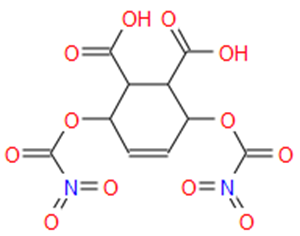
**2.
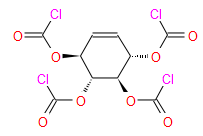
3.
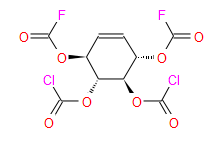


4.
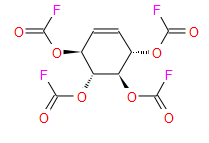
5.
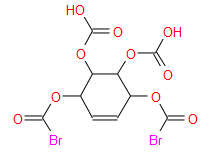
6.
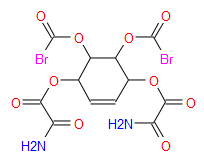


7.
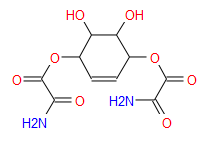
8.
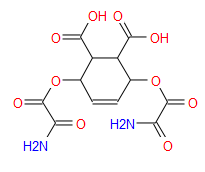
9.
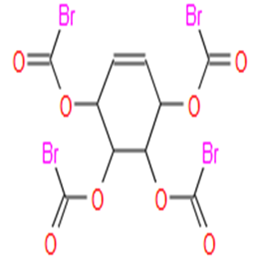


10.
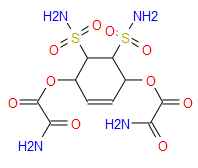


**3. Conduritol C cis-epoxide**

**1.
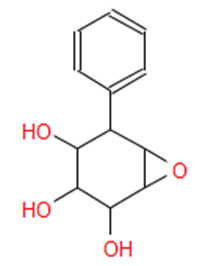
2.**
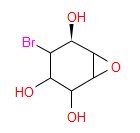
3.
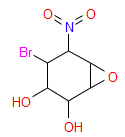
4.
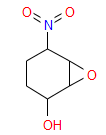


5.
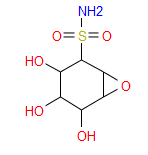
 6.
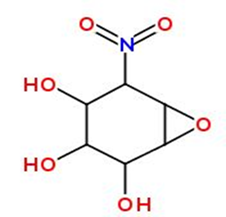
7.
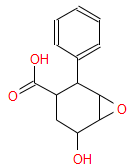


8.
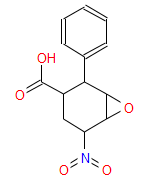
9.
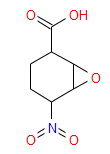
10.
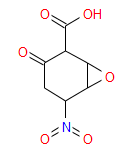


**4. Conduritol D**

**1.
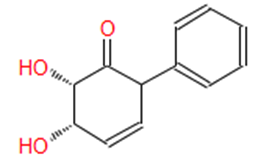
2.**
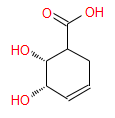
3.
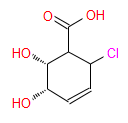
4.
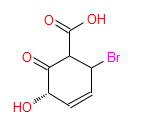


5.
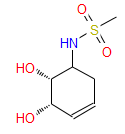
6.
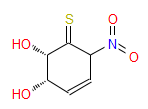
7.
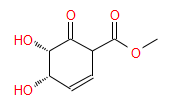


8.
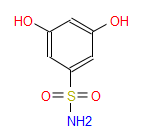
 9.
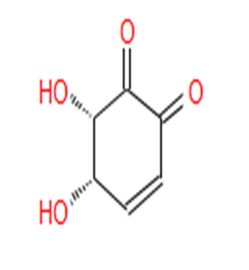
10.
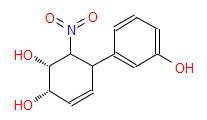


**Figure: Supplementary, S2: 2D structures of the designed analogues.** Framed analogues are selected for the current study.


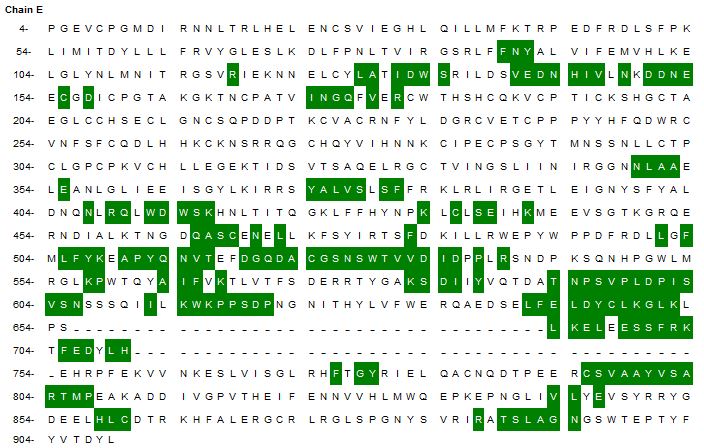


**Figure:** **Supplementary, S3: Active site amino acids of insulin receptor protein 3LOH_E.** Amino acids of active site region. Most potent active site amino acid residues are highlighted in green.


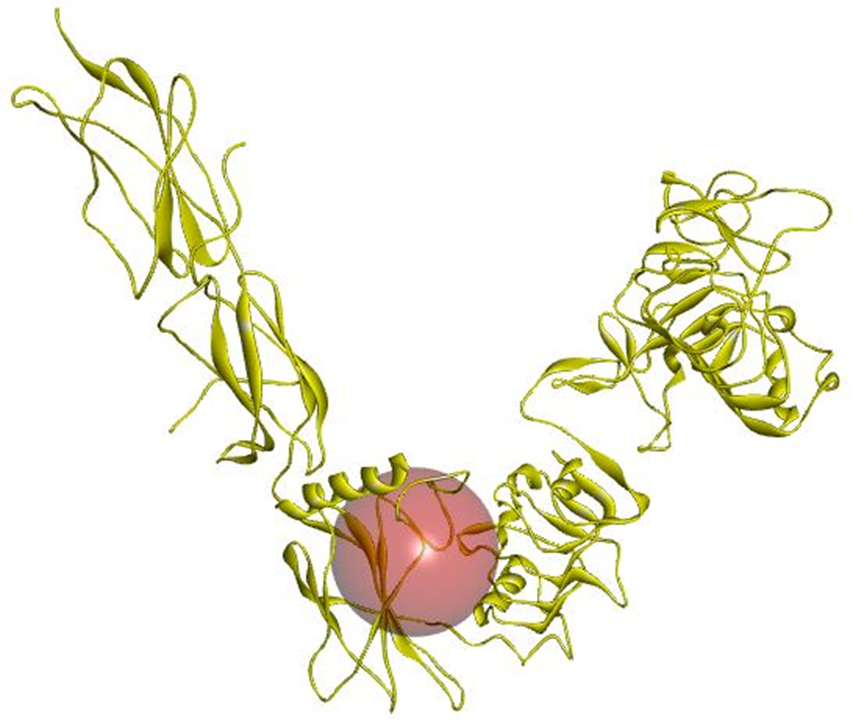


**Figure: Supplementary, S4:** **Binding site of E chain of 3LOH identified by blind docking.**
